# Modulation of mediators derived from whole blood or monocytic cells stimulated with lipopolysaccharide reduces endothelial cell activation

**DOI:** 10.1186/cc10619

**Published:** 2012-03-20

**Authors:** A Schildberger, T Stoifl, D Falkenhagen, V Weber

**Affiliations:** 1 Danube University Krems, Austria

## Introduction

Modulation of inflammatory mediators with specific or selective adsorbents may represent a promising supportive therapy for septic patients. The aims of this study were to modulate mediator concentrations from lipopolysaccharide (LPS)-stimulated whole blood or monocytic THP-1 cells with specific or selective adsorbents and to compare the influence on endothelial cell activation.

## Methods

Whole blood or THP-1 cells (1 × 10^6 ^cells per ml medium containing 10% human plasma) [1] were stimulated with 10 ng/ml LPS from *Escherichia coli *for 4 hours. Mediator modulation was performed with either a specific adsorbent for TNFα which was based on sepharose particles functionalized with anti-TNFα antibodies, or with a selective albumin-coated polystyrene divinylbenzene copolymer (PS-DVB) [2]. Human umbilical vein endothelial cell (HUVEC) activation was monitored for 15 hours by measuring secretion of IL-6 and IL-8, as well as surface expression of the adhesion molecules ICAM-1 and E-selectin.

## Results

Conditioned media derived from whole blood (CMB) or THP-1 cells (CMT) both contained approximately 1,300 pg/ml TNFα which is known to be an important stimulator for HUVEC [1,2]. However, CMB led to a significantly higher HUVEC activation as compared to CMT, as indicated by increased secretion of IL-6 and IL-8 (IL-6: 52,000 vs. 2,000 pg/ml; IL-8: 295,000 vs. 43,000 pg/ml), as well as significantly increased E-selectin surface expression (50 vs. 12 mean fluorescence intensity for CMP and CMT, respectively). Adsorption of inflammatory mediators from the conditioned medium of whole blood or THP-1 cells either with the specific TNFα adsorbent or with the selective PS-DVB beads resulted in decreased endothelial cell activation, as shown by statistically significant reduction of IL-6 and IL-8 secretion from HUVEC, as well as statistically significant reduction of surface expression of the adhesion molecules ICAM-1 and E-selectin. The reduction of HUVEC activation was more pronounced when applying the selective adsorbent showing that the modulation of more than one cytokine is more effective than removing TNFα alone.

## Conclusion

Inflammatory mediator modulation with specific or selective adsorbents reduces endothelial cell activation and thus may support the development of new therapies for sepsis.
